# A Medical Olive Branch

**Published:** 1888-08-18

**Authors:** 


### The Hospital

A Weekly Institutional Journal of

Science, Medicine, Nursing, and Philanthropy.

*For Hospitals, Asylums, Medical Practitioners, Students, Nurses, Families, Parishes, Congregations, and the Charitable Public.*

Vol. IV. No. 99.] [August 18, 1888.

### A Medical Olive Branch.

The British Medical Association deserves well of the public for this reason, if for no other, that it annually gives opportunities for the leading minds in medicine and surgery to expound to their confreres and the world the position and progress of their all-important arts. The traditions of the Association have this year been honorably excelled by the President, Dr. W. T. Gairdner, of Glasgow, Dr. Clifford Allbutt, of Leeds, Dr. William Macewan, and others of the first rank and eminence in their several specialties. Dr. Gairdner, who, by a long life of honest work and intelligent study, has acquired among medical men throughout the world a position of singular respect and authority, upheld the claim of the modern physician to a place among the very highest of the scientific men of the times. Whilst the true physician is a specialist, he is not merely a specialist; whilst he is a learned man, he is not a pedant; whilst he studies disease and its management with the accuracy and impartiality of a passionless scientific temper, he also remembers that each "case" which is brought for his consideration is a living, sentient mortal, strangely compounded of mind and matter, of fear and fortitude; in every respect a creature of "like passions with himself."

The physician and the surgeon of the present day contrast favourably with many of their earlier prototypes. It should not, however, be forgotten that the modern conception of the medical man is not new, but was present to the mind and more or less realised in the conduct of many members of the healing art so long ago as the time of Hippocrates. The ablest of the Greeks at that early period, not only in art and literature, but also in science and medicine, were fully abreast of the moderns in boldness and originality of thought, and in intelligent comprehension of the true methods of scientific progress. Dr. Gairdner renders good service to the cause of intellectual freedom by the clearness of his statements and the soundness of his arguments on this point. That the physicians of the middle ages, and their followers for many decades after the revival of letters, were often hidebound pedants and soulless millhorses, grinding everlastingly the same dry bones of medical maxims and unproved hypotheses, no one denies. But that was not entirely their own fault. The "mephitic vapours" of a blind and tyrannous ecclesiasticism stupefied them, and not them alone, but all the intellect of the nations, with rare and noble exceptions. The typical doctors of physic of Chaucer and Molière are to us, who claim to be the descendants of the worthier types, incomplete as to their manhood and eminently unsatisfactory as professors of their art. That atheism or semi-atheism should have distinguished them on the religious side, whilst a dead and senseless orthodoxy should have been the most marked characteristic of their everyday work—these are features of the medical life of ten or fifteen centuries which we would fain wish had never been visible.

The physicians of those times must undoubtedly bear their full share of blame. But rulers and priests were really the gaolers who locked the doors of the house of knowledge and possessed themselves of the key. An ecclesiastical orthodoxy, blinded with ignorance and pride, was mad enough to think and teach that all truth in heaven and earth had been committed to its keeping. Whoever dared, in any region of intellectual activity, to think differently from what the Church taught, or from what many of its ignorant disciples conceived that it taught—was in danger of being branded as a heretic, and of finding out for himself the full significance of that often honourable, but always dangerous appellation. If, under these circumstances, the doctor confined his researches and his practice to those ways which were established and therefore safe, he only did the same as the cleric, and as every other professional man of his times. We are not approving this safe but contemptible mode of intellectual stasis; we are merely affirming that it was not peculiar to the physician.

Dr. Gairdner is of opinion that there has been a veritable new birth of the medical profession. Its purposes are new, its objects new, its methods new. Tradition has been resolutely flung off. The dead hand has no longer any authority. The physician is now what Hippocrates said he ought always to be, and what his very title implies that he is—a student, a disciple, a learner from Nature. Things, not words—facts, not maxims, are now his chief authorities. This new habit of mind, Dr. Gairdner conceives, will not only make the physician a competent interpreter of the facts of Nature, and a true because an intelligent helper of Nature, but will dispose him to regard with candid and open mind those great and potential facts which to a large extent lie outside the limited sphere of his ordinary avocations, but yet which overlap and force themselves constantly upon his notice. The great facts of mind, of morals, of religion, natural and revealed, have been always supposed to possess little interest for the ordinary man of physic. This general conception of the physician as more than half an atheist has probably been, in a great measure, true. Dr. Gairdner thinks it will not be so true of the future as it has been of a past race of physicians. The average human mind is becoming more comprehensive as its action becomes more free. Religious subjects, too, are now treated with a scientific freedom and candour which would have been regarded as profanity in past times. Not only is the dead hand removed from medicine, but it is also beginning to be removed from ecclesiastical organisations, from books, and from all the traditions of men. Every man claims the right to look and decide for himself. Formerly he often decided without looking, and his decision was of a negative character. He will now look before deciding, with the result that his decision will often be positive rather than negative.

The reconciliation of religion and science has been a dream of open-minded and generous spirits ever since the days of Butler, and long before. Dr. Gairdner, in the spirit of an intelligent believer in the Christian faith, holds that that reconciliation is possible and certain; but only on the condition that the dead hand of a mechanical and unscientific orthodoxy shall be removed from the body of religious dogma, as it has been from the chaotic and hopeless mass of medical tradition. A free human mind is a strong human mind, a mind capable of seeing unity in diversity, purpose in seeming chaos, goodness in apparent evil, conscious rule and just authority everywhere. It is a sign of the times that the latest flag of truce which has appeared between the contending armies of science and faith should have come from the medical ranks. All those who know Dr. Gairdner are well aware that the word of reconciliation spoken by him is no word of mere expediency or unmeaning politeness. It expresses the inner conviction of a scholar, a man of science and a physician, whose whole life has been devoted to the unfaltering search for truth, in every field of activity open to the human mind. Medical men and men of science who incline to materialism and to atheism, will be well advised to attach due authority to that attitude of mind which is so modestly scientific and so nobly devout. And dogmatic theologians, who think that their little systems comprehend the whole of God's universe, and who so readily scorn and condemn those who cannot see with them—they too will do well to stir up in themselves that meek and candid temper which is at once the mark and sign of the true man of science and of the intelligent Christian. Science and medicine in the person of Dr. Gairdner have held out the olive branch. Let us hope that the truce thus begun will end in a reasonable and permanent peace.

